# Development of a CD19 PET tracer for detecting B cells in a mouse model of multiple sclerosis

**DOI:** 10.1186/s12974-020-01880-8

**Published:** 2020-09-18

**Authors:** Marc Y. Stevens, Haley C. Cropper, Katherine L. Lucot, Aisling M. Chaney, Kendra J. Lechtenberg, Isaac M. Jackson, Marion S. Buckwalter, Michelle L. James

**Affiliations:** 1 Department of Radiology, Molecular Imaging Program at Stanford, 1201 Welch Rd, Stanford, CA 94305 USA; 2 Department of Pathology, Stanford University, Stanford, CA USA; 3 Department of Neurology & Neurological Sciences, Stanford University, Stanford, CA USA

**Keywords:** PET, Multiple sclerosis, EAE mice, B cells, CD19

## Abstract

**Background:**

B cells play a central role in multiple sclerosis (MS) through production of injurious antibodies, secretion of pro-inflammatory cytokines, and antigen presentation. The therapeutic success of monoclonal antibodies (mAbs) targeting B cells in some but not all individuals suffering from MS highlights the need for a method to stratify patients and monitor response to treatments in real-time. Herein, we describe the development of the first CD19 positron emission tomography (PET) tracer, and its evaluation in a rodent model of MS, experimental autoimmune encephalomyelitis (EAE).

**Methods:**

Female C57BL/6 J mice were induced with EAE through immunization with myelin oligodendrocyte glycoprotein (MOG_1–125_). PET imaging of naïve and EAE mice was performed 19 h after administration of [^64^Cu]CD19-mAb. Thereafter, radioactivity in organs of interest was determined by gamma counting, followed by ex vivo autoradiography of central nervous system (CNS) tissues. Anti-CD45R (B220) immunostaining of brain tissue from EAE and naïve mice was also conducted.

**Results:**

Radiolabelling of DOTA-conjugated CD19-mAb with ^64^Cu was achieved with a radiochemical purity of 99% and molar activity of 2 GBq/μmol. Quantitation of CD19 PET images revealed significantly higher tracer binding in whole brain of EAE compared to naïve mice (2.02 ± 0.092 vs. 1.68 ± 0.06 percentage of injected dose per gram, % ID/g, *p* = 0.0173). PET findings were confirmed by ex vivo gamma counting of perfused brain tissue (0.22 ± 0.020 vs. 0.12 ± 0.003 % ID/g, *p* = 0.0010). Moreover, ex vivo autoradiography of brain sections corresponded with PET imaging results and the spatial distribution of B cells observed in B220 immunohistochemistry—providing further evidence that [^64^Cu]CD19-mAb enables visualization of B cell infiltration into the CNS of EAE mice.

**Conclusion:**

CD19-PET imaging can be used to detect elevated levels of B cells in the CNS of EAE mice, and has the potential to impact the way we study, monitor, and treat clinical MS.

## Background

Multiple sclerosis (MS) is a chronic, immune-mediated, neurological disease, characterized by the presence of demyelinating lesions in the brain and spinal cord, and progressive physical and cognitive disability [1]. Nearly 1 million people have MS in the USA alone, with 200 new cases diagnosed each week [2]. Although a variety of disease-modifying therapies [3] exist for the most common type of MS, relapsing-remitting MS (RRMS), disease manifestation is highly heterogeneous and treatment response is variable. Current standard-of-care imaging techniques used to clinically diagnose and monitor MS focus on magnetic resonance imaging (MRI) to identify lesions in the central nervous system (CNS). However, this diagnostic approach lacks molecular information regarding an individual’s immune signature in the whole body and brain.

MS has traditionally been viewed as a T cell-mediated disease, but the critical role of B lymphocytes has become increasingly apparent, as demonstrated by the striking success of B cell-depleting therapies in clinical trials. Rituximab and ocrelizumab, which both target the B cell surface antigen CD20, have been shown to dramatically reduce the annualized relapse rate and delay disability progression in RRMS [4]. More recently, newer B cell therapies have been developed that target CD19; a pan-B cell surface marker expressed across a broader range of B cell subsets than CD20 [5]. One such example is inebilizumab [6], which demonstrated favorable safety and tolerability in a phase I MS trial and marked efficacy in neuromyelitis optica (NMO), another immune-mediated CNS disease affecting the spinal cord and optical nerve [7, 8]. Although these promising B cell-targeted therapies exist, there is currently no way to accurately select patients for these treatments or to quantify their effects on B cell load in the CNS and peripheral organs.

Positron emission tomography (PET) has the potential to answer these questions by providing highly specific, quantitative information regarding the in vivo distribution and burden of B cells in the periphery and CNS. However, no gold-standard PET tracer for B cells in MS currently exists. PET tracers for CD19 and CD20 could reveal clinically relevant information regarding different aspects of B cell biology. Recently, our laboratory reported the use of [^64^Cu]-rituximab as an imaging agent in a mouse model of MS, showing that CD20^+^ B cells can be detected in vivo in the CNS of transgenic mice expressing human CD20 [9]. Although this data is encouraging, the therapeutic monoclonal antibody depleted B cells in vivo, limiting the amount of tracer that could be administered, and therefore the overall sensitivity of the method.

A separate clinical study with ^89^Zr-labeled rituximab focused on quantitation of cerebral binding of the radiolabelled antibody in RRMS patients, following pre-dosing with the therapeutic antibody [10]. Radiolabelled rituximab was not detected in the brain parenchyma, possibly due to saturation of binding sites by the isotopically unmodified rituximab administered for therapeutic purposes. Alternatively, the lack of signal could have been due to CD20 not being expressed by plasmablasts and plasma cells known to be present in the CNS of MS patients [11, 12].

In contrast to CD20-targeting tracers, a PET tracer for CD19 would provide a major advantage because of its potential to detect a broader range of B cell subsets, including pro-B cells, antibody-secreting plasmablasts, circulating plasma cells, and a subset of plasma cells in the bone marrow. Importantly, CD19 does not appear to undergo internalization and is not expressed on T cells (whereas CD20 is), making it a more attractive and specific imaging target [11]. Furthermore, tracers targeting CD19 will enable visualization of B cells that escape CD20 therapy, and ultimately assist with patient stratification, dosing, and therapy monitoring.

Herein, we report the development and preclinical characterization of an antibody-based CD19 PET imaging agent. Our goal was to develop a new PET tracer to test the hypothesis that CD19^+^ B cells can be detected in the CNS and periphery of EAE mice.

## Methods

### Animals

All experiments involving animals were completed in accordance with the Stanford Administrative Panel on Laboratory Animal Care (APLAC), which is accredited by the Association for the Assessment and Accreditation of Laboratory Animal Care (AAALAC International). Female C57BL/6 J wild-type mice were purchased from Jackson Laboratories (11 weeks, Jax #00664) and acclimatized for 1 week prior to experiments. Mice were housed in a temperature-controlled environment under a 12-h light/dark schedule with unrestricted access to food/water.

### EAE induction

**Fig. 1:**
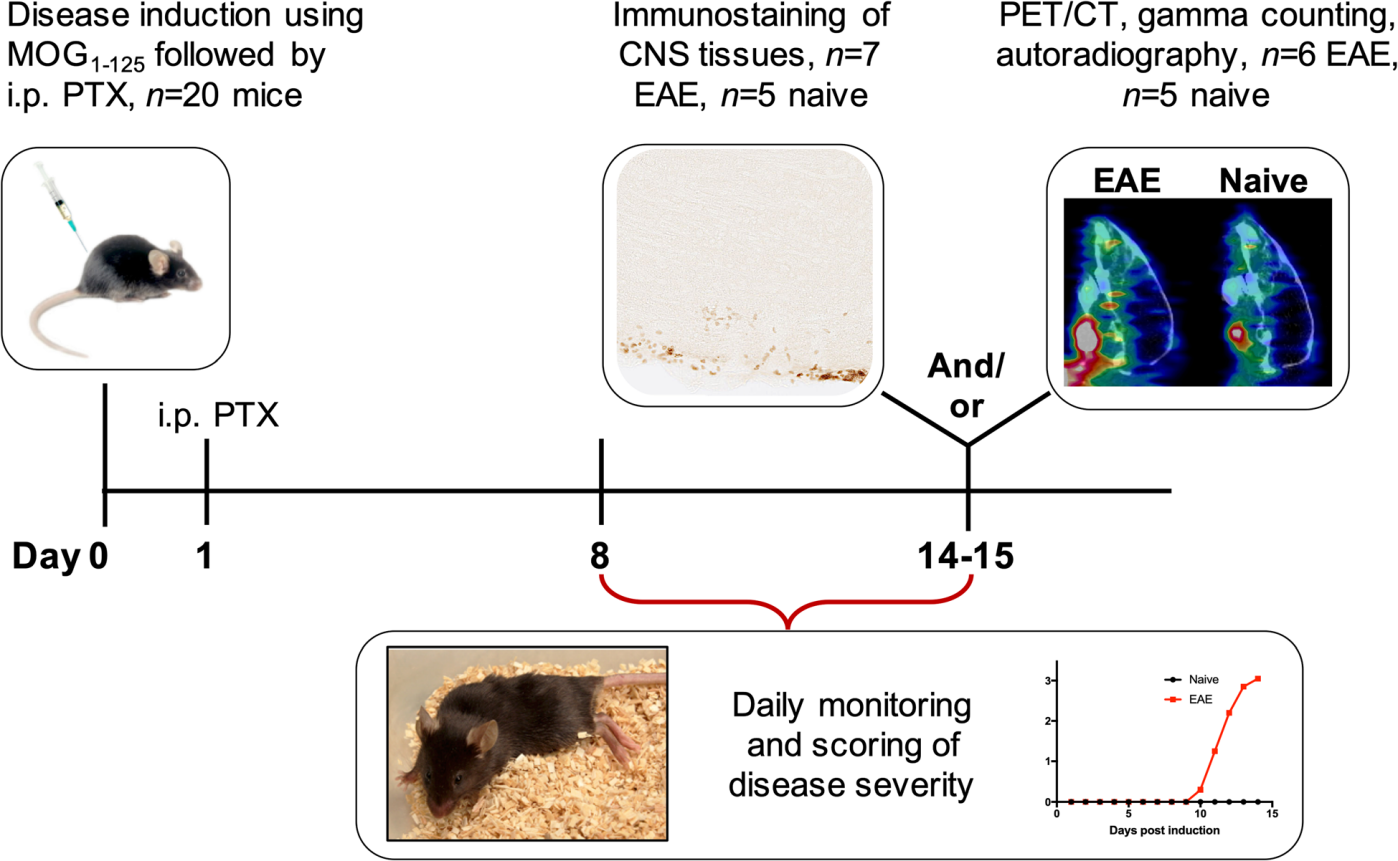
Study design showing timeline of disease induction, immunostaining, PET/CT imaging, and ex vivo analyses. Twenty female C57BL/6 J mice were induced with EAE via subcutaneous administration of myelin oligodendrocyte glycoprotein (MOG_1–125_) and intraperitoneal injection of pertussis toxin (PTX). Mice were subsequently monitored for signs of paralysis from day 8 onwards. On day 14, *n* = 6 mice with hindlimb paralysis (scores 2.5–3.5) and *n* = 5 naive mice were intravenously injected with [^64^Cu]CD19-mAb and imaged using PET/CT 24 h later. The remaining EAE mice were used for in vitro autoradiography/blocking and immunostaining studies

Mice were induced with EAE (Fig. 1) using kits from Hooke Laboratories Inc. (cat# EK2160; Lawrence, MA). After anesthetizing mice with isoflurane gas (2.0–3.0 % for induction and 1.5–2.5 % for maintenance), they were inoculated with myelin oligodendrocyte glycoprotein (MOG_1–125_) in complete Freund’s adjuvant (CFA) (0.2 mL per mouse, S.C.), followed by two pertussis toxin (PTX) injections (80–100 ng per injection, I.P., 2 and 24 h post-MOG injection, Supp. Fig. 1). All mice were weighed and scored daily in the morning from day 8 onwards according to standard EAE scoring systems: 0, no paralysis; 1, loss of tail tone; 2, hind limb weakness or paresis; 3, hind limb paralysis; 4, complete hind leg and partial front leg paralysis. EAE mice were given supplemental food and fluids as necessary.

### Immunostaining

Anti-CD45R (B220) immunostaining of brain tissue from *n* = 7 EAE (of which *n* = 3 mice had not been previously imaged using [^64^Cu]CD19-mAb) and *n* = 5 naïve mice was carried out to investigate the load and location of CNS-infiltrating B cells. Following perfusion with 30 mL of phosphate-buffered saline (PBS), each brain was dissected, and the right hemisphere was fixed for 24 h in 4% paraformaldehyde (PFA) phosphate buffer and stored in 30% sucrose in PBS prior to sectioning at 40 μm (Microm HM 450 sliding microtome; Thermo Scientific). Immunostaining of free-floating brain sections was performed using standard procedures [8]. Briefly, sections were thoroughly washed in tris-buffered saline (TBS) and pretreated with 0.6% H2O2 in 0.3% TritonX-TBS prior to overnight 4 °C incubation with 1:500 biotinylated anti-B220/CD45R antibody (BD Pharmigen) in 0.3% Triton X-TBS. The following day, tissues were incubated for 1 h with the Vectastain Elite ABC kit (Vector Laboratories) then exposed to 0.05% 3,3-diaminobenzidine (Sigma) in 0.1 MTrisCl (pH 7.4) with 0.03% H2O2 for 3-4 min. Lastly, sections were washed in 0.1 M TrisHCl prior to coverslipping.

### DOTA conjugation

Mouse CD19 monoclonal antibody (CD19-mAb) (115502, clone 6D5, Biolegend; San Diego, CA) was conjugated with DOTA-NHS (Macrocyclics; Plano, TX) using metal-free buffers and previously published procedures [12]. Average number of chelator molecules per antibody was determined to be 2–3 using electrospray ionization mass spectrometry (ESI-MS) and matrix-assisted laser desorption/ionization-time of flight (MALDI-TOF).

### [^64^Cu]-radiolabeling

Radiolabeling of DOTA-CD19-mAb (Suppl. Fig. 1) was performed by modifying previously reported general copper labeling methods [8, 13]. Briefly, immunoconjugate (80 ± 11 μg, *n* = 4) in NH_4_OAc (pH 5.5) was added to [^64^Cu]CuCl_2_ (0.06 GBq) in NH_4_OAc under gentle agitation at 37 °C (final volume 100 μL) and the reaction monitored via radioTLC until a labeling efficiency of > 99% was observed (15–30 min). Thereafter, EDTA (50 mM) was added (2 μL). Reactions were analyzed by radioTLC (iTLC-SG glass microfibre strips developed in 50 mM EDTA solution for 3 min) and SEC-HPLC (size-exclusion HPLC, Phenomenex 00H-2146-K0, 5 μm SEC-s3000 400 Å, 300 × 7.8 mm). Pure fractions (> 99%) of [^64^Cu]CD19-mAb were combined and diluted with saline. Prior to in vivo studies, in vitro autoradiography was performed to assess specificity of [^64^Cu]CD19-mAb using mouse spleen and brain tissue (Suppl. Fig. 2).

### In vivo PET/CT acquisition

On day 13 after EAE induction, mice were imaged using a dual microPET/computed tomography (CT) scanner (Inveon, Siemens). Mice (*n* = 6 EAE, clinical score ≥ 3, *n* = 5 naïve) were anesthetized with isoflurane gas and intravenously injected with [^64^Cu]CD19-mAb (4.85–6.88 MBq, equivalent to 6.47–9.30 μg immunoconjugate). CT images were acquired as previously described [13] for anatomical reference prior to a 10 min static PET acquisition 21–24 h post tracer administration. On each imaging day, a calibration factor was calculated using a 20 mL syringe containing a known amount of radioactive [^64^Cu]. PET data was acquired and reconstructed using previously described methods [8].

### PET analysis and quantitation

**Fig. 2:**
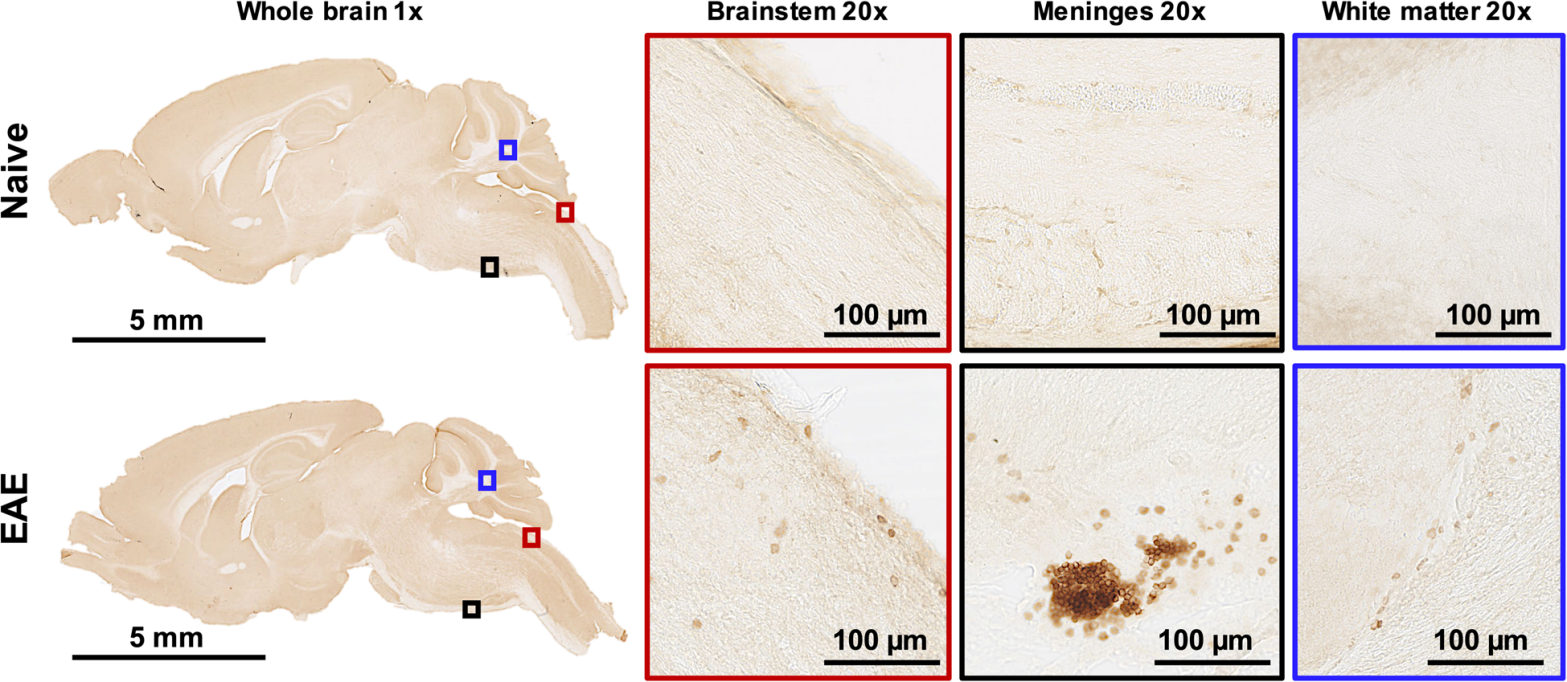
B cell infiltrates are present in brainstem, meninges, and white matter of EAE mice. Representative B220 immunostaining of naïve and EAE mouse brain tissue (*n* = 7 EAE, *n* = 5 naïve mice, average of 4 slices per animal). Scale bars are 5 mm in low magnification (×1) sagittal brain images and 100 μm in high magnification (×20) images of the brainstem, meninges, and cerebellar white matter

CT and PET files were co-registered and analyzed using the VivoQuant 4 (inviCRO) and Inveon Research Workspace 2.5 (Siemens) software to quantify tracer uptake in specific regions and to generate images, respectively. Brain regions of interest (ROIs) were chosen on the basis of our immunohistochemistry staining results (Fig. 2), as well as previous reports of tertiary lymphoid organs in brainstem and meninges of EAE mice [14]. For brain region quantitation, a 3D mouse brain atlas with ROIs for medulla, pons, and white matter was fitted to the PET/CT images via alignment of the atlas with the skull of each mouse (as determined by CT). Quantification of PET signal in all 15 brain regions that are part of the atlas are shown in Suppl. Fig. 3A. PET signal in the femur was quantified to assess levels of CD19^+^ B cells in bone marrow, whereby signal from bone was identified using Otsu thresholding following our previously published procedures [13], after which manual ROIs were drawn for bone marrow. Values were displayed as percentage of injected dose per gram (%ID/g), allowing comparison with gamma counting results.

### Gamma counting and autoradiography

Tissues were collected for gamma counting and autoradiography 24 h after injection of [^64^Cu]CD19-mAb as previously described [8]. Tissues were collected for both in vitro. Briefly, 200–300 μL of blood was collected from the left cardiac ventricle of anesthetized mice prior to perfusion with 30–40 mL of PBS. Left brain hemisphere, heart, liver, spleen, cervical/thoracic (C/TSC), and lumbar (LSC) spinal cord were harvested, weighed, and counted using an automatic gamma counter (Hidex Oy, Helsinki, Finland), allowing %ID/g to be calculated for each organ of interest. Autoradiography was performed using 40 μm-thick left-brain hemisphere sections and whole C/TSC and LSC to obtain high-resolution images of tracer distribution in CNS tissues, expressed as mean pixel intensity/decay-corrected dose (MPI/dose). Brain section anatomy was confirmed by Nissl staining of the same sections as previously described [15]. Autoradiography ROIs (*n* = 4 per group) were drawn on the basis of immunohistochemical findings and were defined as hindbrain (brainstem and cerebellum) and cerebellar white matter (Suppl. Fig. 3B).

### Statistics

Statistical analyses were performed using Prism 7 (GraphPad). Data are represented as mean ± SEM with individual values plotted on bar graphs. Shapiro-Wilk normality tests were conducted prior to unpaired Student’s *t* tests for PET, ex vivo tissue gamma counting (brain, blood, femur, spleen), and ex vivo autoradiography data. Two-way ANOVA with Šídák’s multiple-comparisons post hoc tests were used for ex vivo gamma counting of vertebrae and spinal cord tissues. Pearson’s correlation was used for IHC, autoradiography, and PET correlations. A *p* value of less than 0.05 was considered statistically significant.

## Results

### Disease induction

EAE was induced by subcutaneous injection of MOG_1-125_ in female C57BL/6 J mice to evaluate the utility of [^64^Cu]CD19-mAb for in vivo detection of B cells in a murine model of MS. Peak disease severity (clinical score > 3) was observed within 12–14 days, with clinical scores ranging from 3 to 3.5 reflecting hindlimb paralysis (Fig. 1).

### Mouse model/immunohistochemistry

To verify B cell infiltration into the CNS of immunized mice, B220 immunostaining of brain tissue from *n* = 5 naïve and *n* = 9 EAE mice was performed (Fig. 2). Immunohistochemistry revealed increased B220^+^ cell numbers in the cerebellar white matter and associated meninges (2.46 ± 0.369 vs. 0.409 ± 0.138 cells/mm^2^, **p = 0.00210), as well as in brainstem and associated meninges (5.42 ± 1.65 vs. 0.199 ± 0.0980 cells/mm^2^, *p = 0.0390), particularly in the subarachnoid space, of EAE compared to naïve mice. B cells were found in dense clusters similar to follicle-like structures observed in the meninges of MS patients, with a highly heterogeneous spatial distribution [16].

### Synthesis and characterization of [^64^Cu]CD19-mAb

An average conjugation of 3 DOTA per CD19-mAb was achieved and subsequent radiolabeling with ^64^Cu yielded > 99% radiochemical purity and a molar activity of 2.2 GBq/μmol (*n* = 14 reactions, Suppl Fig. 4, Suppl Fig. 5). Specificity of [^64^Cu]CD19-mAb was evaluated in vitro, whereby blocking results showed significantly decreased tracer binding in naïve spleen and EAE brain tissue that was pre-incubated with 1000-fold mass dose of unlabeled unconjugated mAb (Suppl. Fig. 2).

### PET/CT imaging

**Fig. 3:**
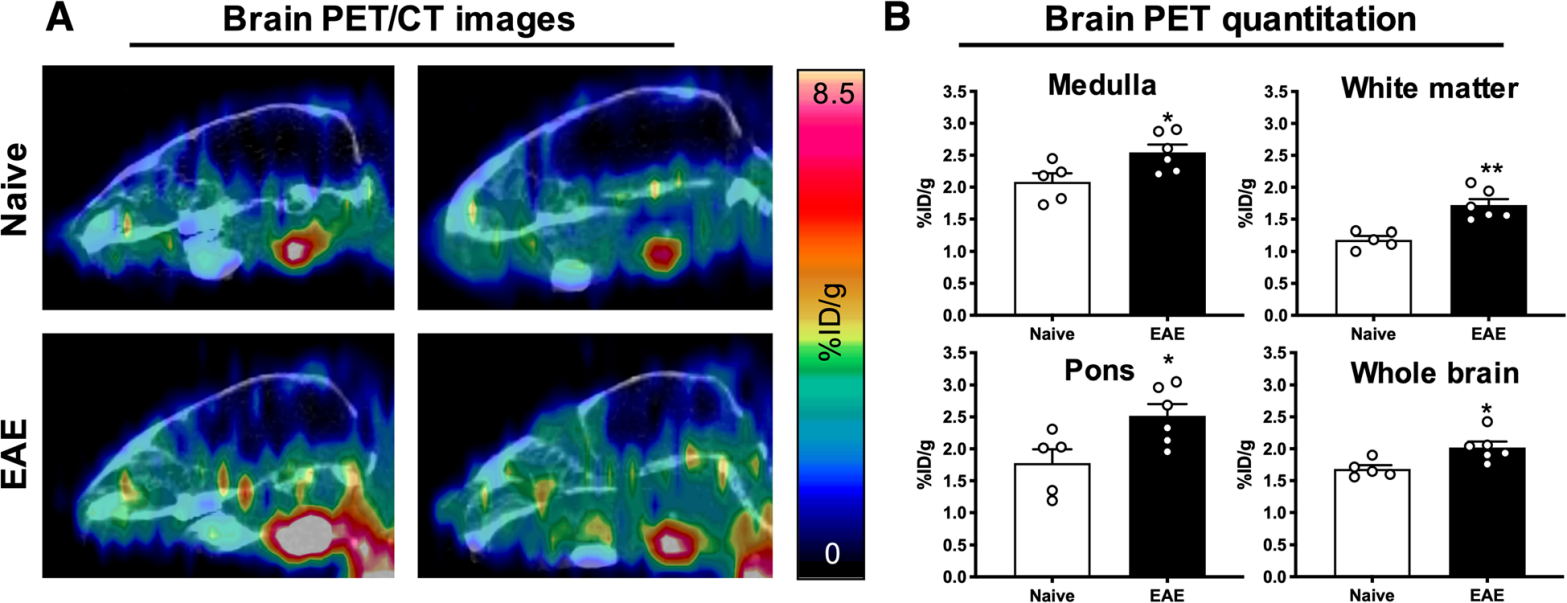
CD19-PET imaging shows increased signal in the brain of EAE mice. **a** Representative sagittal brain PET/CT images of naïve and EAE mice 24 h after [^64^Cu]CD19-mAb injection. **b** Quantitation of tracer binding in medulla, pons, white matter, and whole brain, *n* = 5 per group. *p = < 0.05, **p = < 0.01

To investigate the ability of [^64^Cu]CD19-mAb to detect CD19^+^ B cells in immunized mice, in vivo PET images were acquired and quantified in regions implicated in EAE pathology (medulla, pons, white matter, and whole brain) for *n* = 6 EAE and *n* = 5 naïve mice (Fig. 3a). PET quantitation of white matter (1.72 ± 0.0950 vs. 1.18 ± 0.0590 %ID/g, **p = 0.00140) and whole brain (2.02 ± 0.0920 vs. 1.68 ± 0.0600 %ID/g, *p = 0.0173) showed significant increases in tracer uptake for EAE compared to naïve mice (Fig. 3b). This pattern was also observed between EAE and naïve mice in the pons (2.52 ± 0.183 vs. 1.78 ± 0.220 %ID/g, *p = 0.0270) and medulla (2.54 ± 0.123 vs. 2.08 ± 0.135 %ID/g, *p = 0.0310, Fig. 3b). Conversely, tracer uptake in femur showed reduced binding of [^64^Cu]CD19-mAb in bone marrow of EAE compared to naïve mice (4.18 ± 0.366 vs. 6.76 ± 0.957 %ID/g, *p = 0.0242, Suppl. Fig. 6).

### Ex vivo biodistribution and autoradiography

**Fig. 4:**
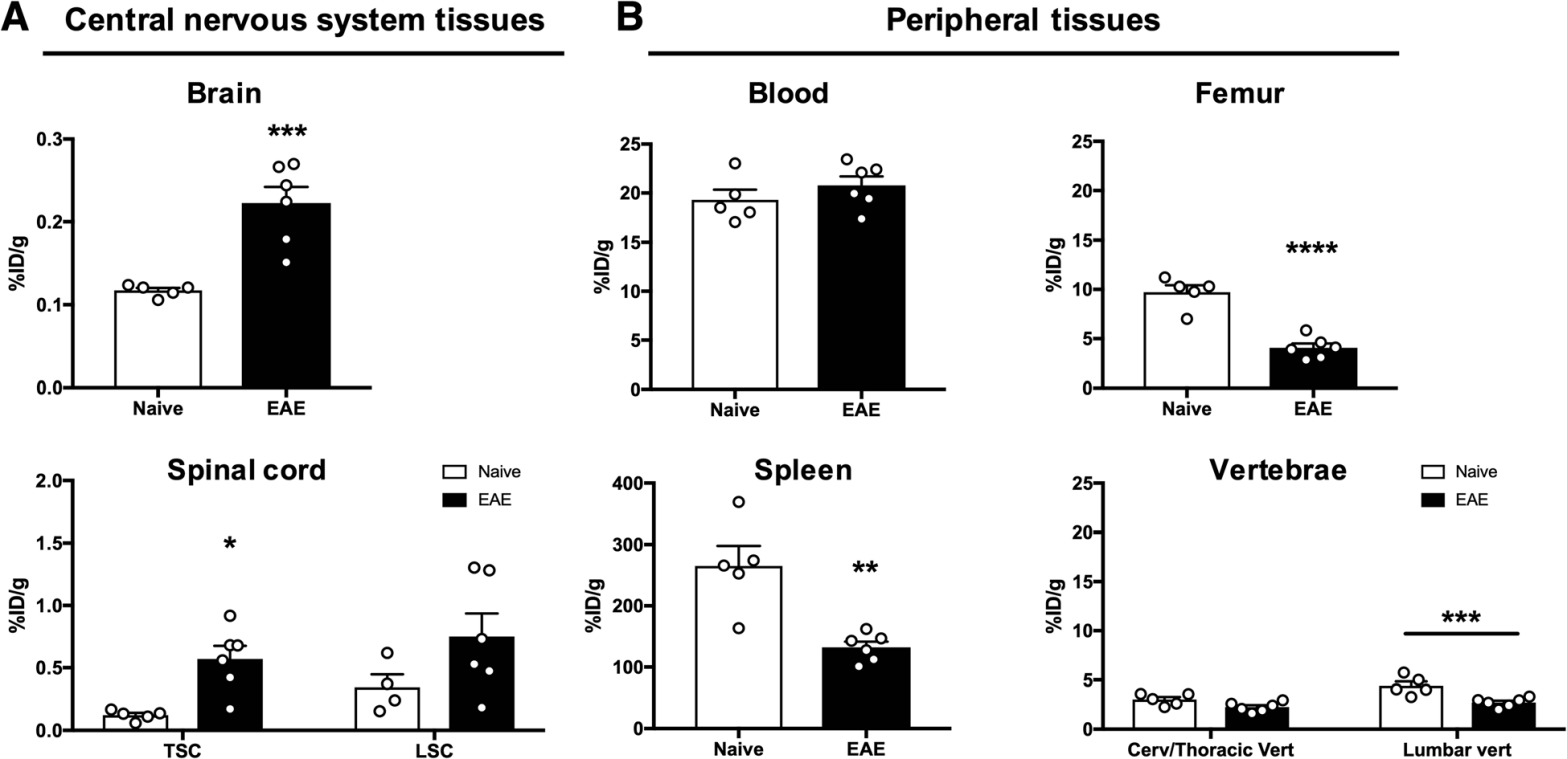
Ex vivo biodistribution confirmed in vivo PET findings. Ex vivo gamma counting of [^64^Cu]CD19-mAb biodistribution in (**a**) central nervous system and (**b**) peripheral tissues 24 h after tracer injection, *n* = 5 per group, *p = < 0.05, **p = < 0.01, ***p = < 0.001, ****p = < 0.0001

To compare tracer uptake in tissues of EAE and naïve mice without the contribution of tracer in the blood pool, ex vivo gamma counting was performed after perfusion using half brains, cervical/thoracic spinal cord, lumbar spinal cord, blood (from cardiac puncture prior to perfusion), femur, heart, liver, muscle, spleen, cervical/thoracic vertebrae, and lumbar vertebrae (Fig. 4 and Suppl. Fig. 7). In the CNS (Fig. 4a), tracer uptake was significantly elevated in the brain (0.220 ± 0.0200 vs. 0.120 ± 0.00300 %ID/g, *p* = 0.001) and cervical/thoracic spinal cord (0.571 ± 0.104 vs. 0.122 ± 0.0180 %ID/g, *p* = 0.0375 of EAE compared to naïve mice, with a trend in the lumbar spinal cord (0.750 ± 0.186 vs. 0.350 ± 0.0780 %ID/g, *p* = 0.0661). Separating the vertebrae from spinal cord tissue revealed a significant decrease in tracer uptake in the lumbar vertebrae (2.73 ± 0.188 vs. 4.42 ± 0.429 %ID/g, *p* = 0.0007), but not in the cervical/thoracic vertebrae (2.26 ± 0.198 vs. 3.02 ± 0.262 %ID/g, *p* = 0.121) between EAE and naïve mice. EAE mice exhibited significantly lower tracer uptake in femur (4.07 ± 0.450 vs. 9.71 ± 0.717 %ID/g, *p* < 0.0001) and spleen (132 ± 9.31 vs. 265 ± 32.7 %ID/g, *p* = 0.002) compared to naïve mice (Fig. 4b). However, there were no significant differences in tracer uptake in the muscle (1.52 ± 0.18 vs. 1.09 ± 0.04 %ID/g, *p* = 0.0650), liver (10.2 ± 1.16 vs. 8.08 ± 0.56 %ID/g, *p* = 0.15), or heart (1.71 ± 0.16 vs. 1.38 ± 0.204 %ID/g, *p* = 0.240) between EAE and naïve mice (Suppl. Fig. 7).

**Fig. 5:**
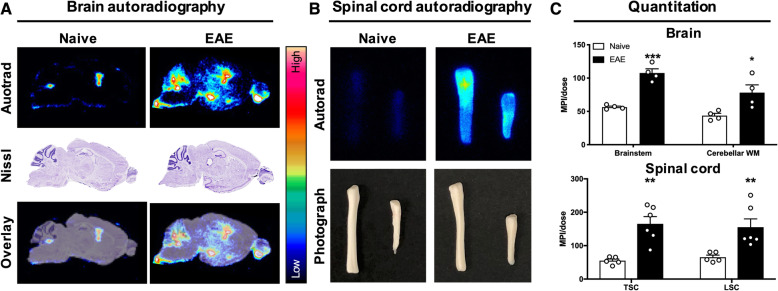
High-resolution ex vivo autoradiography of brain sections and spinal cord tissue further confirmed in vivo PET results. Ex vivo autoradiography (autorad) of (**a**) 40 μm-thick sagittal brain sections and (**b**) dissected whole spinal cords—cervical/thoracic (left) and lumbar (right). Nissl staining and photographs are shown for brain sections and spinal cords respectively. (**c**) Quantitation of radiotracer signal in brainstem, cerebellar white matter (WM), and spinal cord regions, expressed as mean pixel intensity divided by decay-corrected dose injected (MPI/dose); *n* = 4 per group for brainstem analysis (since brainstem was not successfully dissected for all mice), *n* = 5 per group for spinal cord analysis. *p = < 0.05, **p = < 0.01, ***p = < 0.001

Brain and spinal cord tissues were further evaluated using ex vivo autoradiography to determine the spatial distribution of tracer uptake in the CNS with higher resolution (Fig. 5 and Suppl. Fig 3B). Quantitation revealed significantly increased tracer uptake in the brainstem (107.7 ± 6.291 vs. 56.44 ± 1.532 MPI/dose, *p* = 0.0002), cerebellar white matter (78.2 ± 11.6 vs. 43.7 ± 3.37 MPI/dose, *p* = 0.0290), whole cerebellum (73.2 ± 7.44 vs. 45.7 ± 3.03 MPI/dose, *p* = 0.0172), cervical/thoracic spinal cord (165 ± 21.6 vs. 55 ± 5.06 MPI/dose, *p* = 0.0011), and lumbar spinal cord (155 ± 24.9 vs. 65.3 ± 6.39 MPI/dose, *p* = 0.0060) of *n* = 4 EAE compared to *n* = 4 naïve representative mice (Fig. 5c and Suppl. Fig. 3B).

### Correlation between immunohistochemistry and imaging findings

**Fig. 6:**
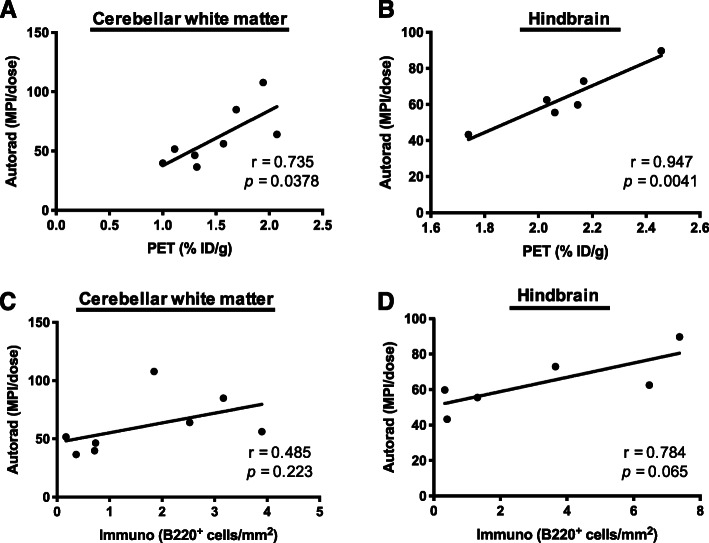
Ex vivo autoradiography correlates with PET or B220 immunohistochemistry. Correlation between ex vivo autoradiography and PET or B220 immunohistochemistry of (A/C) cerebellar white matter and (B/D) cerebellum + brainstem (i.e., hindbrain), naïve, and EAE mice (*n* = 3–4 per group)

To better understand how [^64^Cu]CD19-mAb tracer signal relates to levels of B cells in individual mice, we investigated the correlation between B220 immunohistochemistry, autoradiography, and PET quantitation. Results depicted in Fig. 6a illustrate a significant positive correlation between in vivo PET and ex vivo autoradiography of [^64^Cu]CD19-mAb in cerebellar white matter, (*r* = 0.735, *p = 0.0378) and hindbrain (brainstem and cerebellum) (*r* = 0.947, **p = 0.00410). Correlation between autoradiography and B220 staining in the cerebellar white matter revealed a lack of correspondence between ex vivo imaging and B cell counts (*r* = 0.485, *p* = 0.223, Fig. 6c), whereas hindbrain autoradiography showed a trending positive correlation with B cell numbers (*r* = 0.784, *p* = 0.0650, Fig. 6d).

## Discussion

In this study, we sought to generate a PET tracer specific for CD19-positive B cells to enable detection of a broad range of B cell subsets in vivo, including pro B cells, plasmablasts, and early plasma cells. We successfully synthesized a ^64^Cu-labeled mAb targeting CD19 and demonstrated its utility for visualizing B cell distribution in EAE mice with high sensitivity, in both the CNS and peripheral tissues.

The selected EAE model using full-length MOG_1–125_ protein was well-suited for this work due to its ability to induce B cell immune responses [17]. In this EAE model, a sub-population of antigen-activated B cells differentiate into plasma cells, resulting in the production of autoantibodies against MOG. Anti-MOG autoantibodies produced by B cells result in CNS inflammation and subsequent demyelination [17–19]. Moreover, previous reports have shown that induction of EAE using MOG_1–125_ in mice leads to B cell infiltration into the spinal cord and brain [8]. Indeed, our B220 immunostaining confirmed the existence of follicle-like structures containing dense B cell aggregates in the meninges, in addition to B cell infiltration in the brainstem and cerebellar white matter, of EAE mice, compared to negligible levels of B cells in naïve mouse brain tissue (Suppl. Fig. 8). These results are in agreement with investigations of MS lesions from treatment-naïve patients showing CD20^+^ B cell infiltration in all disease stages [20]. In addition to the presence of B cells in MS CNS lesions, they often form follicle-like clusters in extra-parenchymal regions, including the subarachnoid space within the meninges [21], which we also observed in our diseased mice. The contribution of demyelination to EAE pathogenicity has been previously demonstrated by Al-Ani and coworkers, who showed a positive immunohistochemical correlation between B cell infiltration in the spinal cord with demyelination markers [22]. Although the chief focus of the current study was to validate the in vivo binding properties of our PET tracer, future studies will involve investigating the relationship between demyelination markers and CD19-PET.

Prior to initiating in vivo studies, we first verified the specificity of our CD19 PET tracer by performing in vitro autoradiography both with and without pre-blocking using excess CD19-mAb. The marked decrease in autoradiography signal in brain and spleen tissue from EAE mice after blocking confirmed specific binding of [^64^Cu]CD19-mAb to CD19^+^ cells and warranted its further use and evaluation. Subsequently, in vivo PET findings revealed increased signal in the brainstem and white matter of EAE mice, mirroring the spatial distribution observed in B220 immunohistochemical staining of brain tissue from the same cohort of mice. It is important to note that while the contribution of blood signal (i.e., tracer residing in blood pool) increases the observed %ID/g for in vivo brain PET imaging, these results were corroborated by quantitative ex vivo gamma counting following perfusion, in which significantly increased signal was observed in whole brain of EAE mice (after the removal of blood). Additionally, high-resolution autoradiography enabled detailed analysis of the location of PET tracer binding in CNS tissues, and matched the distribution pattern of B220^+^ cells observed by brain immunohistochemistry. Autoradiography was especially useful for verifying [^64^Cu]CD19-mAb binding in the white matter which is difficult to discern using PET alone due to the limited spatial resolution of small animal PET. Similarly, autoradiography was used to demonstrate elevated tracer binding in spinal cord tissue from EAE mice compared to that observed in naïve mice. Unfortunately, the limited spatial resolution of small animal PET imaging and issues regarding the partial volume effect makes it difficult to delineate in vivo tracer binding to CD19+ cells in the spinal cord versus tracer binding to CD19+ cells in the bone marrow of the vertebral column (which are two relatively small structures in close proximity). Therefore, in vivo mouse PET images obtained during this study do not allow an accurate visualization of B cells in spinal cord, due to signal spillover from the vertebral bone marrow into the spinal cord and vice versa. Importantly, this will not be an issue in the clinical setting since these structures are significantly larger in humans.

Tracer binding was also quantified in tissues outside the CNS via PET imaging and ex vivo gamma counting. Visually, we observed higher PET signal in lymph nodes of EAE mice compared to naives (Suppl. Fig. 9). For bone marrow, the pattern of binding was the reverse of what was observed in lymph nodes and in the CNS—i.e., a dramatic reduction in signal in both femur and lumbar vertebrae from EAE compared to naïve mice. This may be due to trafficking of B cells out of repositories such as bone marrow in tandem with disease progression. Similarly, gamma counting of spleens revealed an almost 50% reduction in signal from EAE animals, reflecting a possible egress of B cells during the diseased state. A previously reported longitudinal flow cytometric analysis of EAE mouse tissues [23] shows that both T and B cells traffic from the spleen to the lymph nodes where they become activated, proliferate, and subsequently travel through the circulation to the CNS. This phenomenon may also occur in MS patients. In fact, the commonly prescribed MS therapeutic, fingolimod, exerts much of its beneficial effects by reducing the egress of lymphocytes out of secondary lymphoid tissues, decreasing numbers of T and B cells in circulation [24]. Although outside the scope of the current study, a method to track live cells emanating from the bone marrow lineage following adoptive transfer in EAE mice will be valuable. A recent study by Ortega and coworkers [25] used B cells labeled with a fluorophore in a stroke model, allowing tracking from the periphery into the CNS. Our future investigations will employ a similar approach, wherein mice lacking lymphocytes will be seeded with B cells and induced with EAE prior to sequential imaging, allowing monitoring of B cell reservoirs and their involvement in CNS pathogenicity via longitudinal PET imaging.

Finally, we sought to investigate the relationship between observed in vivo and ex vivo tracer signal and underlying B cell load, as well as the association between in vivo and ex vivo imaging data. A strong positive correlation was observed between in vivo PET and ex vivo autoradiography in the hindbrain, providing further evidence of tracer specificity. A positive trend could also be seen between autoradiography of the hindbrain and increasing B cell count, reflective of the disease state. However, this trend was only weakly discernible when individual brain regions were investigated, possibly due to heterogeneous spatial distribution of B cell clusters across the brain similar to that observed in the clinical setting. This approach is potentially limited by the use of opposing brain hemispheres for immunohistochemistry and autoradiography. This could be mitigated by using adjacent sections for the separate techniques, with the possible risk of compromised tissue quality for B220 staining. Additionally, the expression of B220 (CD45R), a pan-B cell marker in mice, does not overlap fully with that of CD19, and cells negative for either surface marker would be excluded from this analysis.

## Conclusion

Overall, the results from the current work provide strong evidence that a tracer targeting CD19 can detect B cells in the CNS and periphery of a mouse model of MS. The increasing focus on B cells as therapeutic targets in neurodegenerative diseases such as MS, and more recently NMO [7], highlights the need for methodology that will allow treatment monitoring and patient selection for anti-B cell clinical trials. The tools developed as part of this work have the potential to inform future preclinical research by providing a new tool to visualize B cells in the context of different diseases. Our future studies will focus on evaluating this CD19 PET tracer in mouse models of other neurological and neuromuscular diseases involving B cells, including stroke [26], NMO [27], and myasthenia gravis [28], in addition to lymphoma. We will also investigate the use of ^89^Zr-labeled CD19 mAbs, allowing imaging at later timepoints with a reduced contribution from the blood pool. Although the investigation of individual subsets of CD19+ B cell populations and their pathogenic effects was outside of the scope of this investigation, we acknowledge the importance of understanding which B cell subsets contribute to the observed PET signal in different parts of the body and CNS in the context of health and disease, and therefore this will also be part of additional planned studies.

## Supplementary information

**Additional file 1: Suppl. Fig 1.** Generation of the first reported PET tracer for CD19+ B cells via DOTA-conjugation of a CD19-specific mAb and subsequent radiolabeling with copper-64 ([^64^Cu]). **Suppl. Fig. 2. In vitro autoradiography of [**^**64**^**Cu]CD19-mAb and anatomical staining of spleen and brain tissue sections.** Frozen tissue sections were allowed to reach ambient temperature, after which they were washed in 50 mM Tris-HCl for 3x5 min at pH 7.4, before drying under airflow for 30 min. A perimeter was drawn around the tissue with a hydrophobic pen and blocking antibody (1000x based on specific activity calculated by spectrophotometry) was added to half of the slides, followed by incubation for 30 min at ambient temperature. After washing off the blocking slides (3x2 min Tris-HCl, 50 mM, pH 7.4), a solution containing [^64^Cu]CD19-mAb tracer (500 μL, 2.5 μCi/mL) was carefully applied using a pipette and slides were left to incubate for 30 min, before 3x2 min washes in Tris-HCl (50 mM, pH 7.4, ambient temperature). Finally, all slides were dipped twice into ice water and left to dry for 30 min under airflow. Slides were transferred to a phosphorimaging plate and stored at -20 °C for 24 h, before development using a Typhoon laser scanner. Gel images were analyzed using ImageJ software. **Fig. 3A. In vivo PET detects increased signal in distinct brain regions**. 3D brain atlas tool from Invicro (Boston, MA) allows automated analysis of individual brain regions. Skull CT was used to define total brain volume for determination of activity. *n*=5 naïve, *n*=6 EAE, **p=.0082, two-way ANOVA. **Suppl. Fig. 3B. Uptake of [**^**64**^**Cu]CD19-mAb corresponds to spatial distribution observed in immunohistochemistry.** A. Ex vivo autoradiography, 50 μm resolution. B. Quantitation of uptake in cerebellum, n=4 naïve and EAE, *p=.0286, unpaired t-test. Coloured outlines denote ROIs drawn in ImageJ. **Suppl. Fig. 4A**. Samples were analyzed by ESI-MS on an Agilent 1260 HPLC and Bruker MicroTOF-Q II, running a 12 min gradient of 5 to 95% 0.1% formic acid in MeCN, solvent A was 0.1% formic acid in water. Panels A and C depict the TIC of unconjugated and immunoconjugate (2.9 DOTA) running in positive mode, B and D depict the deconvoluted mass spectra of the same compounds. The column was a Waters MassPREP 5x2.1mm diphenyl desalting column, the temperature was 50 °C, and the flow rate was 0.3ml/min. Injection volume was 15 μL. **Suppl. Fig. 4B**. Antibody samples underwent buffer exchange/desalting using Zip-Tip C_18_ filters, at a final concentration of approx. 1 mg/mL in 0.1% TFA/60% MeCN/40% H_2_O. Each spot was loaded with approx. 1 μg, 0.5 pmol internal standard (BSA) was added and samples were analysed using an Applied Biosystems SCIEX 5800 TOF/TOF instrument with high mass detection. **A**. Unconjugated antibody. **B**. Immunoconjugate following addition of chelator. **Suppl. Fig. 5**. Antibody samples were analyzed by SEC-HPLC. A) UV (280 nm), unconjugated CD19 anti-mouse antibody, clone 6D5. B) UV (280 nm), immunoconjugate following DOTA chelation. C. Radiochromatogram of B. **Suppl. Fig. 6. Femur PET/CT shows increased signal in naïve mice compared to EAE, likely due to CD19-positive B cells trafficking out of bone marrow in diseased mice.** Bone was identified using Otsu thresholding and bone marrow ROIs were drawn in manually. A) Representative PET/CT image from femur of naïve and EAE mice. (B) In vivo PET quantitation of PET image, *n*=5 naïve, *n*=6 EAE, **p=.0071, unpaired t-test. **Suppl. Fig. 7. Ex vivo gamma counting of [**^**64**^**Cu]CD19-mAb biodistribution showing no significant differences between EAE and naive heart, liver, and muscle 24 h after tracer injection.** *n*=5 naïve, *n*=6 EAE,**p=.0021, ****p<.0001, unpaired t-test. **Suppl. Fig. 8. Quantitative immunohistochemical quantitation of B220**^**+**^ **cell load in cerebellum and brainstem** *n*=5 naïve, *n*=10 EAE,*p=.0195 for cerebellum, *p=.0417 for brainstem, unpaired t-test. Brainstem was missing in one EAE mouse following tissue preparation and sectioning (*n*=9). **Suppl. Fig. 9.** PET/CT images acquired 20 h post-injection, showing uptake of tracer in a) cervical lymph node, b) spleen, c) mesenteric lymph node and d) inguinal lymph node. Percentage of injected dose per gram (%ID/g) scaled to allow comparison between different mice.

## Data Availability

The datasets during and/or analyzed during the current study will be made available from the corresponding author on reasonable request.

## Abbreviations

AAALAC International: Association for the assessment and accreditation of laboratory animal care
APLAC: Administrative panel on laboratory animal care
Autorad: Autoradiography
CFA: Complete Freund’s adjuvant
CNS: Central nervous system
CT: Computed tomography
C/TSC: Cervical/thoracic spinal cord
EAE: Experimental autoimmune encephalomyelitis
ESI-MS: Electrospray ionization mass spectrometry
i.p.: Intraperitoneal
i.v.: Intravenous
LSC: Lumbar spinal cord
mAb: Monoclonal antibody
MALDI-TOF: Matrix-assisted laser desorption/ionization-time of flight
MOG: Myelin oligodendrocyte glycoprotein
MPI/dose: Mean pixel intensity/decay-corrected dose
MRI: Magnetic resonance imaging
MS: Multiple sclerosis
NMO: Neuromyelitis optica
O.C.T.: Optimal cutting temperature
PBS: Phosphate buffered saline
PET: Positron emission tomography
PFA: Paraformaldehyde
PTX: Pertussis toxin
ROI: Region of interest
RRMS: Relapsing-remitting MS
TBS: Tris-buffered saline
WM: Cerebellar white matter
%ID/g: Percentage of injected dose per gram
