# Supplementary material for: Development of a CD19 PET tracer for detecting B cells in a mouse model of multiple sclerosis
Source: J Neuroinflammation. 2020 Sep 18;17:275. doi: 10.1186/s12974-020-01880-8 (PMC7501720; doi:10.1186/s12974-020-01880-8)
Supplement: Supplementary file 1 — Additional file 1: Suppl. Fig 1. Generation of the first reported PET tracer for CD19+ B cells via DOTA-conjugation of a CD19-specific mAb and subsequent radiolabeling with copper-64 ([64Cu]). Suppl. Fig. 2. In vitro autoradiography of [64Cu]CD19-mAb and anatomical staining of spleen and brain tissue sections. Frozen tissue sections were allowed to reach ambient temperature, after which they were washed in 50 mM Tris-HCl for 3x5 min at pH 7.4, before drying under airflow for 30 min. A perimeter was drawn around the tissue with a hydrophobic pen and blocking antibody (1000x based on specific activity calculated by spectrophotometry) was added to half of the slides, followed by incubation for 30 min at ambient temperature. After washing off the blocking slides (3x2 min Tris-HCl, 50 mM, pH 7.4), a solution containing [64Cu]CD19-mAb tracer (500 μL, 2.5 μCi/mL) was carefully applied using a pipette and slides were left to incubate for 30 min, before 3x2 min washes in Tris-HCl (50 mM, pH 7.4, ambient temperature). Finally, all slides were dipped twice into ice water and left to dry for 30 min under airflow. Slides were transferred to a phosphorimaging plate and stored at -20 °C for 24 h, before development using a Typhoon laser scanner. Gel images were analyzed using ImageJ software. Fig. 3A. In vivo PET detects increased signal in distinct brain regions. 3D brain atlas tool from Invicro (Boston, MA) allows automated analysis of individual brain regions. Skull CT was used to define total brain volume for determination of activity. n=5 naïve, n=6 EAE, **p=.0082, two-way ANOVA. Suppl. Fig. 3B. Uptake of [64Cu]CD19-mAb corresponds to spatial distribution observed in immunohistochemistry. A. Ex vivo autoradiography, 50 μm resolution. B. Quantitation of uptake in cerebellum, n=4 naïve and EAE, *p=.0286, unpaired t-test. Coloured outlines denote ROIs drawn in ImageJ. Suppl. Fig. 4A. Samples were analyzed by ESI-MS on an Agilent 1260 HPLC and Bruker MicroTOF-Q II, running a 12 m [file 12974_2020_1880_MOESM1_ESM.docx]

**Development of a CD19 PET tracer for detecting B cells in a mouse model of multiple sclerosis**

Marc Y. Stevens^1^^, Haley C. Cropper^1^^, Katherine L. Lucot^2^, Aisling M. Chaney^1^, Kendra J. Letchenberg^3^, Isaac M. Jackson^1^, Marion S. Buckwalter^3^, Michelle L. James^1,3^*.

*^1^Department of Radiology, Molecular Imaging Program at Stanford, CA.*

*^2^Department of Pathology, Stanford University, CA.*

*^3^Department of Neurology & Neurological Sciences, Stanford University, CA*

* To whom correspondence should be addressed: [mljames@stanford.edu](mailto:mljames@stanford.edu)

^ Equal contribution

##

## Radiochemistry

**Suppl. Fig 1.** Generation of the first reported PET tracer for CD19+ B cells via DOTA-conjugation of a CD19-specific mAb and subsequent radiolabeling with copper-64 ([^64^Cu]).

## In vitro autoradiography of EAE tissues

**Spleen (Naive)**


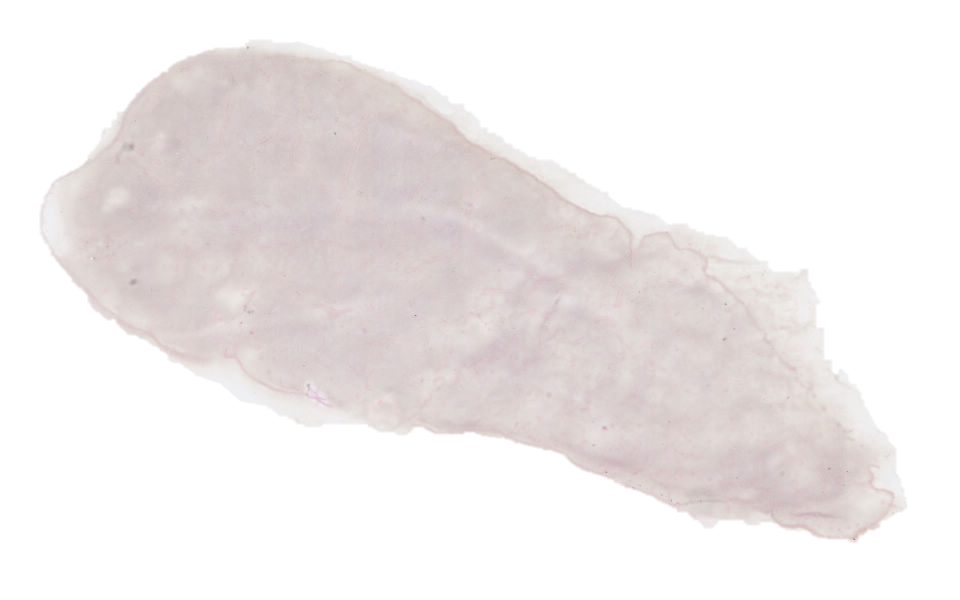


**Blocking (1000x)**


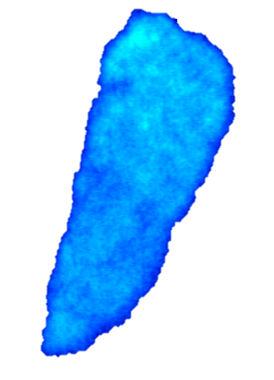

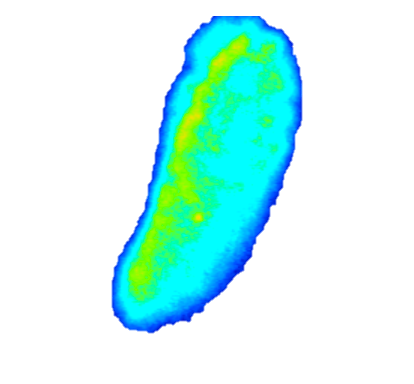

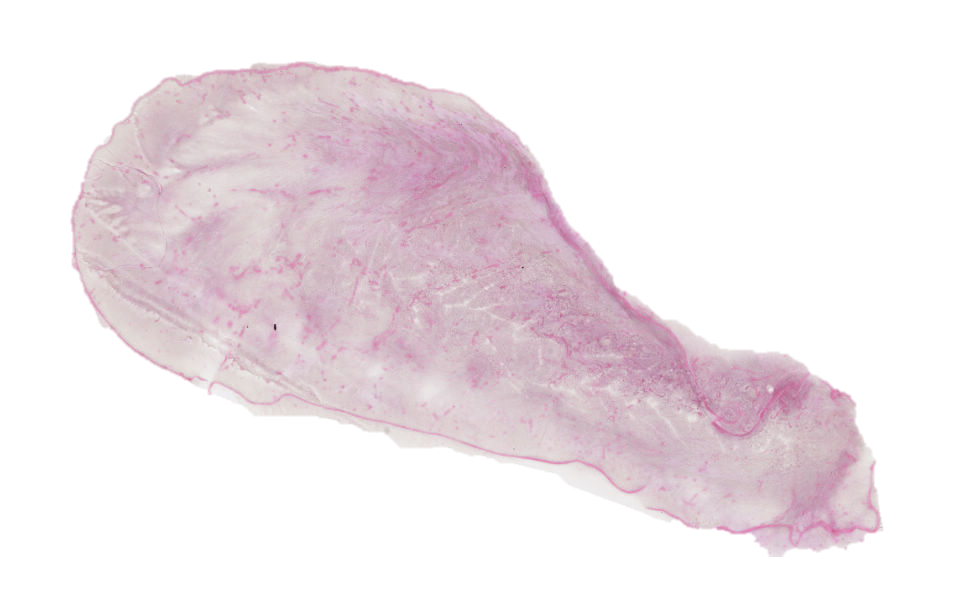

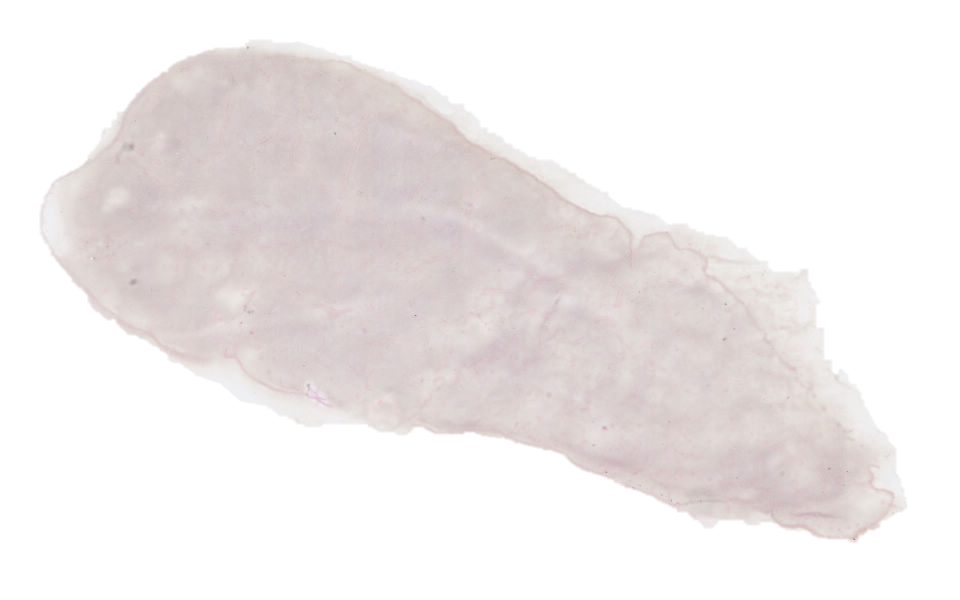

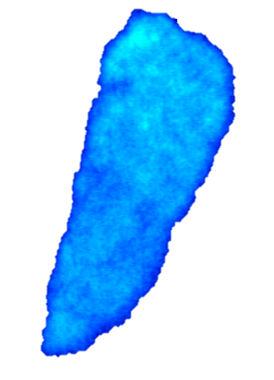

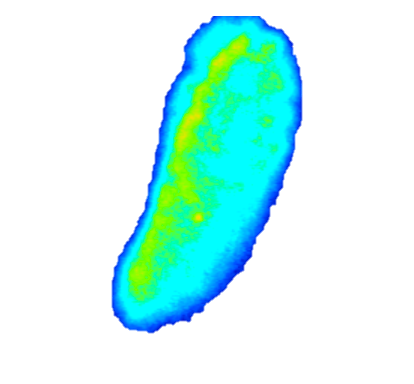

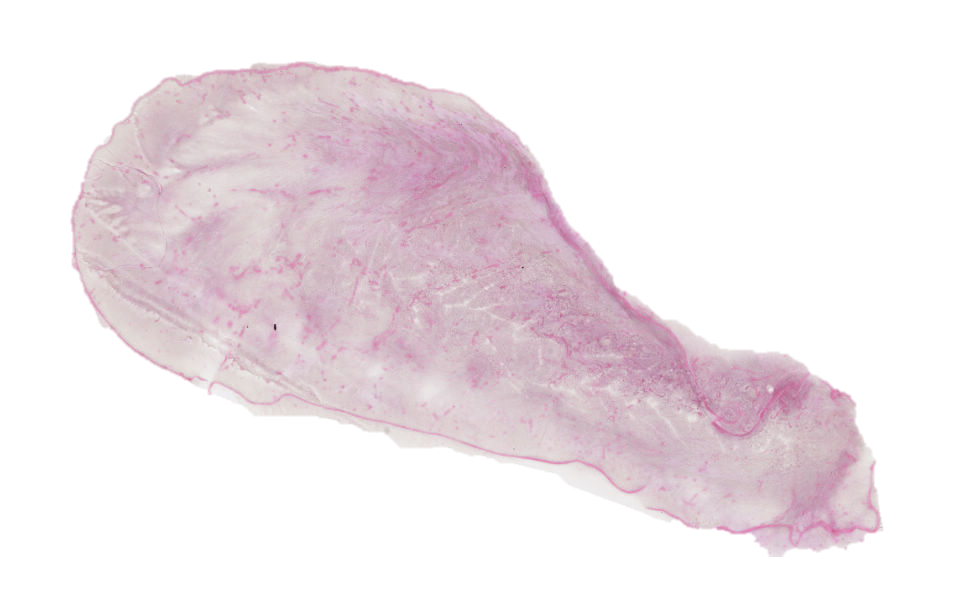


**ARG**

**H&E**

**Overlay**


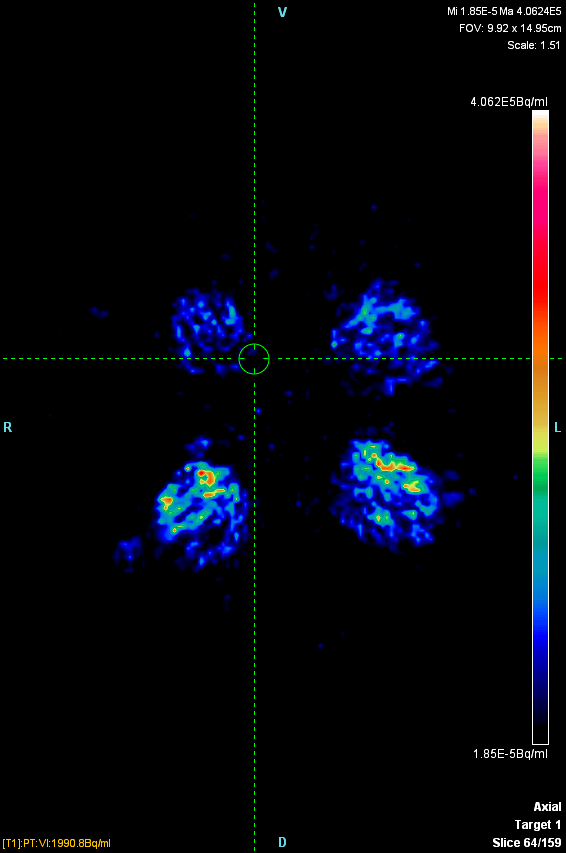


High

Low

**Brain (EAE)**

**Blocking (1000x)**


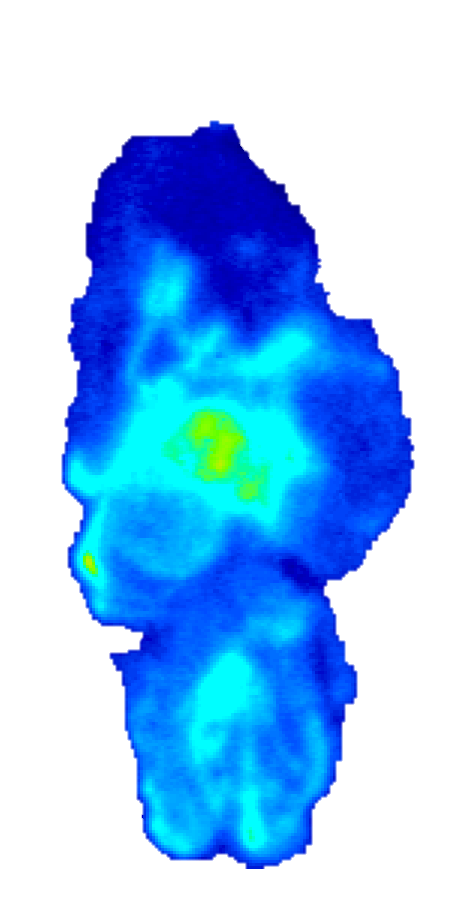

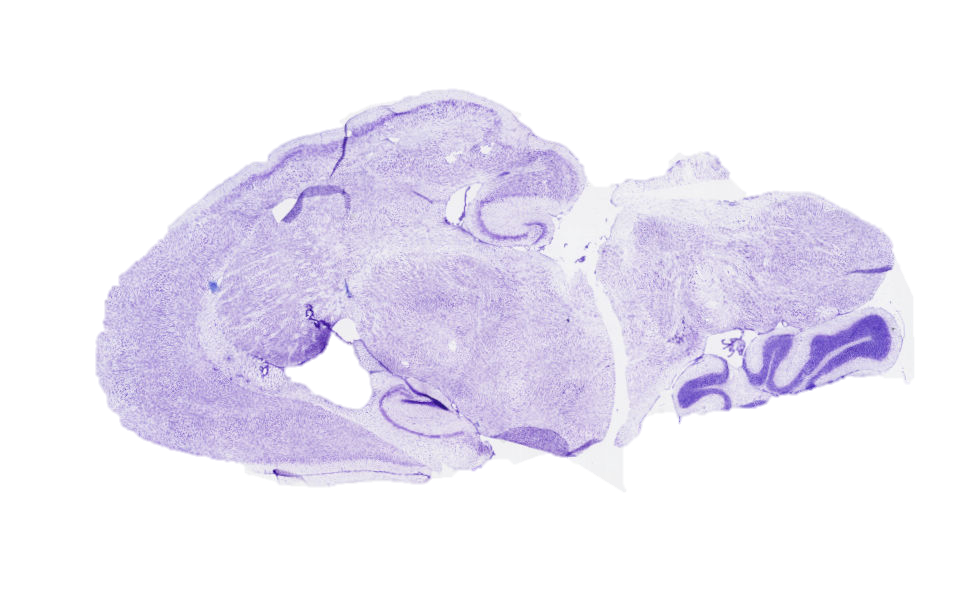

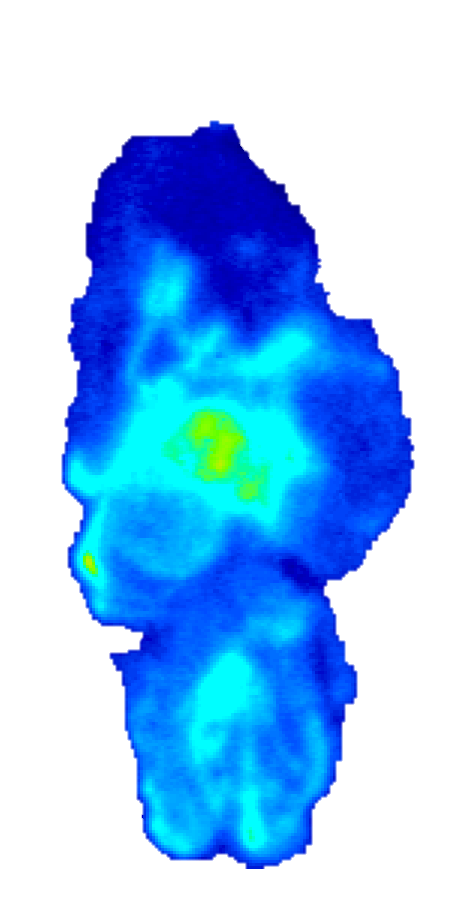

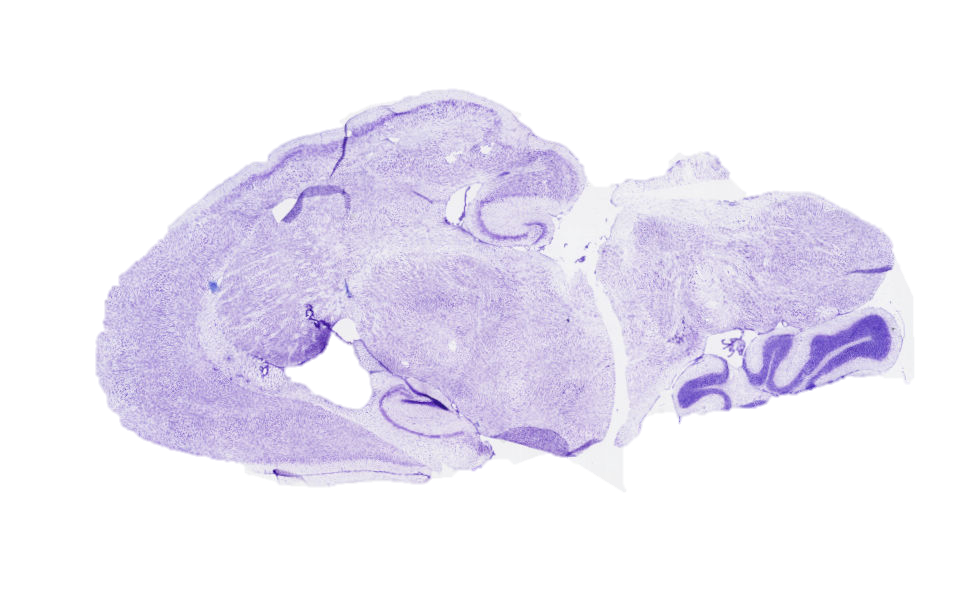

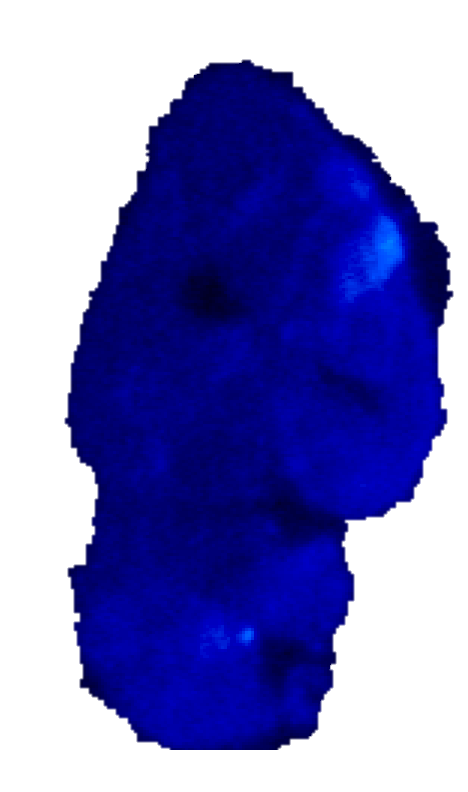

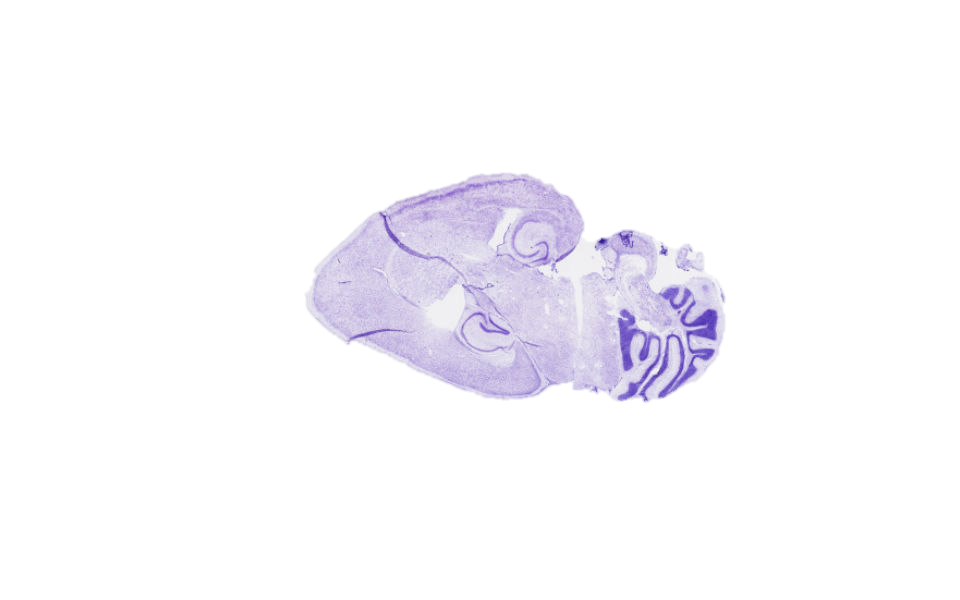

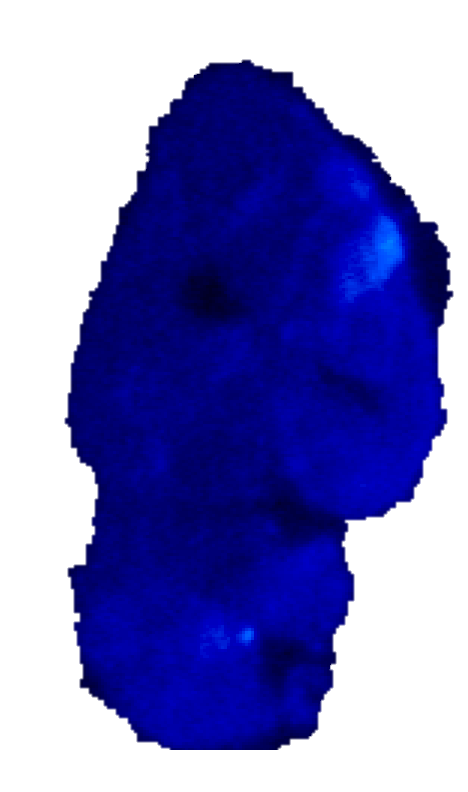

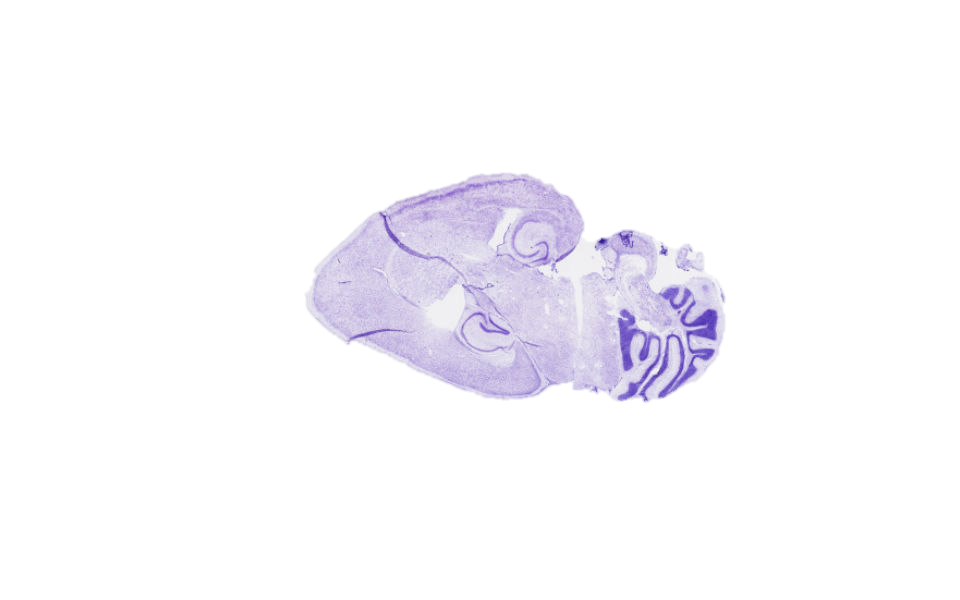


**ARG**

**Nissl**

**Overlay**

**Suppl. Fig. 2. In vitro autoradiography of [^64^Cu]CD19-mAb** **and anatomical staining of spleen and brain tissue sections.** Frozen tissue sections were allowed to reach ambient temperature, after which they were washed in 50 mM Tris-HCl for 3x5 min at pH 7.4, before drying under airflow for 30 min. A perimeter was drawn around the tissue with a hydrophobic pen and blocking antibody (1000x based on specific activity calculated by spectrophotometry) was added to half of the slides, followed by incubation for 30 min at ambient temperature. After washing off the blocking slides (3x2 min Tris-HCl, 50 mM, pH 7.4), a solution containing [^64^Cu]CD19-mAb tracer (500 μL, 2.5 μCi/mL) was carefully applied using a pipette and slides were left to incubate for 30 min, before 3x2 min washes in Tris-HCl (50 mM, pH 7.4, ambient temperature). Finally, all slides were dipped twice into ice water and left to dry for 30 min under airflow. Slides were transferred to a phosphorimaging plate and stored at -20 °C for 24 h, before development using a Typhoon laser scanner. Gel images were analyzed using ImageJ software.

## In vivo PET brain atlas and ex vivo autoradiography ROIs

**Suppl. Fig. 3A. In vivo PET detects increased signal in distinct brain regions**. 3D brain atlas tool from Invicro (Boston, MA) allows automated analysis of individual brain regions. Skull CT was used to define total brain volume for determination of activity. *n*=5 naïve, *n*=6 EAE, ***p*=.0082, two-way ANOVA.

**Suppl. Fig. 3B. Uptake of [^64^Cu]CD19-mAb corresponds to spatial distribution observed in immunohistochemistry.** A. Ex vivo autoradiography, 50 μm resolution. B. Quantitation of uptake in cerebellum, n=4 naïve and EAE, **p*=.0286, unpaired t-test. Coloured outlines denote ROIs drawn in ImageJ.

## ESI-MS and MALDI-TOF spectra

**A**

**B**

**C**

**D**

**Suppl. Fig. 4A**. Samples were analyzed by ESI-MS on an Agilent 1260 HPLC and Bruker MicroTOF-Q II, running a 12 min gradient of 5 to 95% 0.1% formic acid in MeCN, solvent A was 0.1% formic acid in water. Panels A and C depict the TIC of unconjugated and immunoconjugate (2.9 DOTA) running in positive mode, B and D depict the deconvoluted mass spectra of the same compounds. The column was a Waters MassPREP 5x2.1mm diphenyl desalting column, the temperature was 50 °C, and the flow rate was 0.3ml/min. Injection volume was 15 μL.

**Internal standard**

**Internal standard**

**A**

**B**

**Suppl. Fig. 4B**. Antibody samples underwent buffer exchange/desalting using Zip-Tip C_18_ filters, at a final concentration of approx. 1 mg/mL in 0.1% TFA/60% MeCN/40% H_2_O. Each spot was loaded with approx. 1 μg, 0.5 pmol internal standard (BSA) was added and samples were analysed using an Applied Biosystems SCIEX 5800 TOF/TOF instrument with high mass detection. **A**. Unconjugated antibody. **B**. Immunoconjugate following addition of chelator.

## HPLC-SEC chromatograms of non-radiolabeled and radiolabeled CD19-mAb


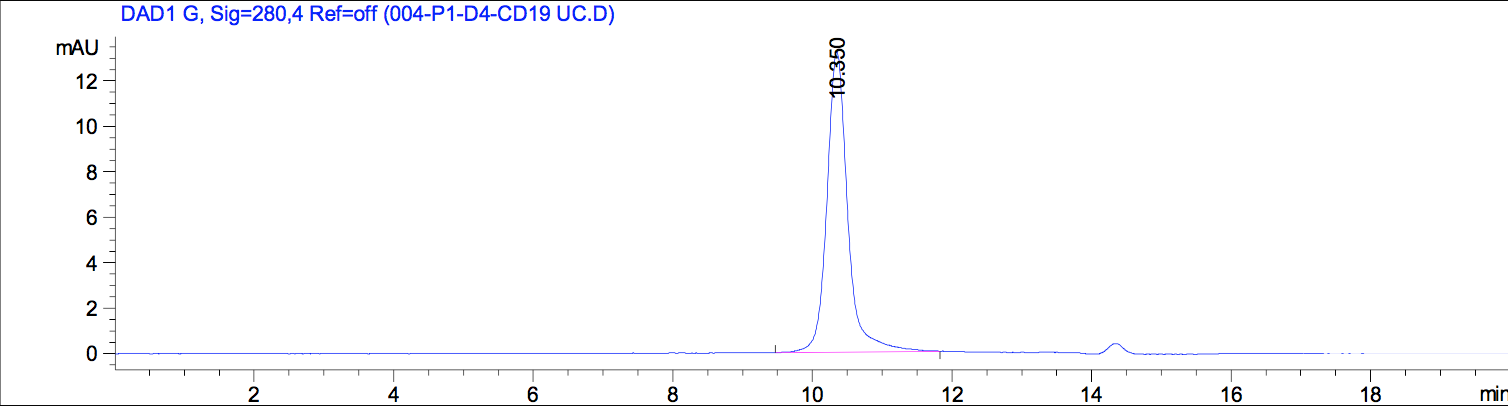


**A**


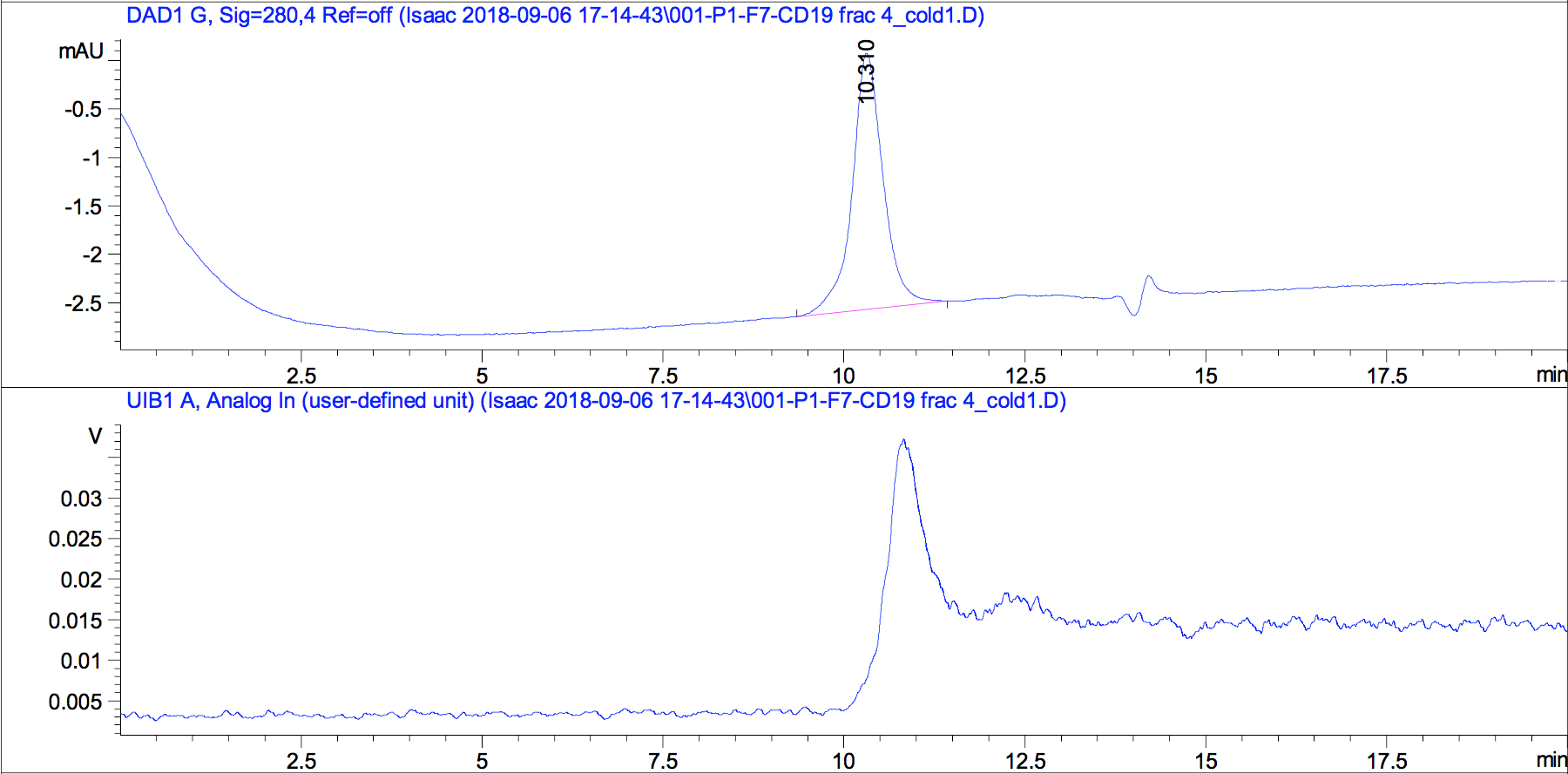


**B**

**C**

**Suppl. Fig. 5**. Antibody samples were analyzed by SEC-HPLC. A) UV (280 nm), unconjugated CD19 anti-mouse antibody, clone 6D5. B) UV (280 nm), immunoconjugate following DOTA chelation. C. Radiochromatogram of B.

## In vivo femur PET images and quantitation


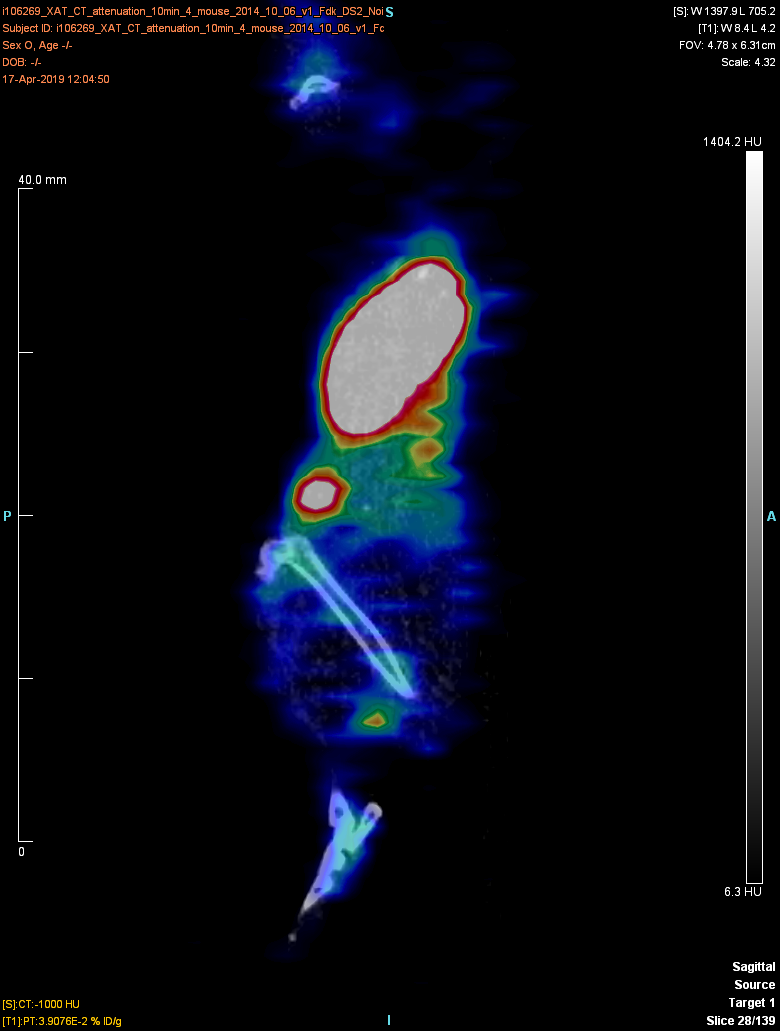

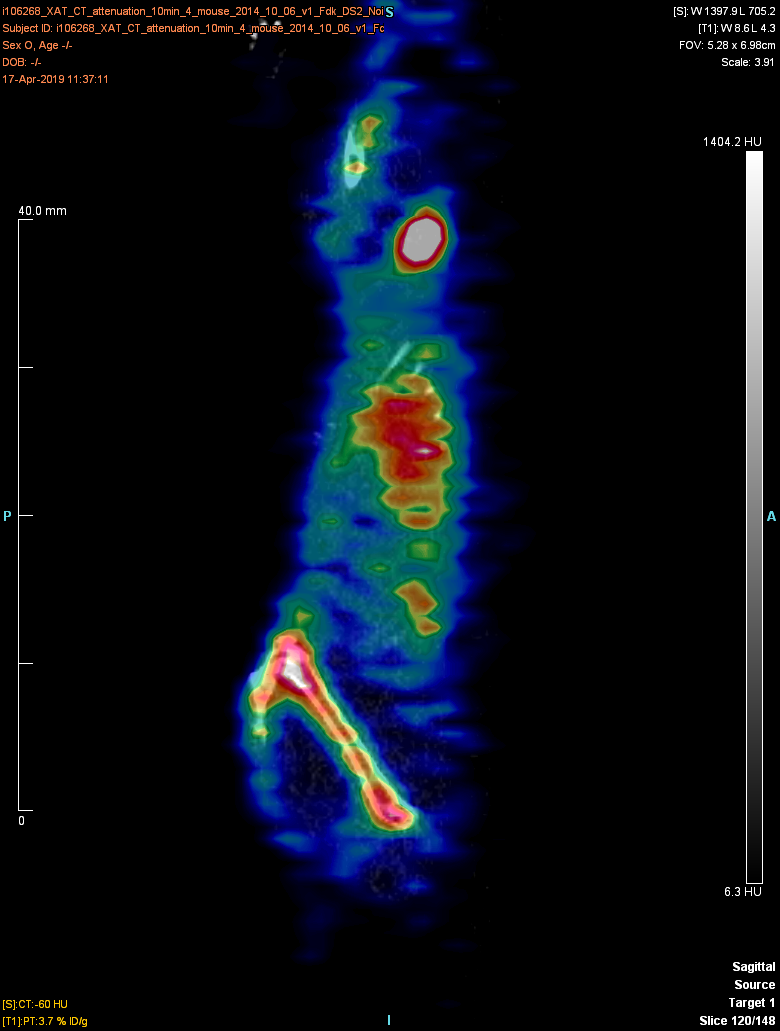


**Naive**

**EAE**

**A**

**B**

**Suppl. Fig. 6. Femur PET/CT shows increased signal in naïve mice compared to EAE, likely due to CD19-positive B cells trafficking out of bone marrow in diseased mice.** Bone was identified using Otsu thresholding and bone marrow ROIs were drawn in manually. A) Representative PET/CT image from femur of naïve and EAE mice. (B) In vivo PET quantitation of PET image, *n*=5 naïve, *n*=6 EAE, **p=.0071, unpaired t-test.

## Ex vivo gamma counting of peripheral tissue

**Muscle**

**Liver**

**Heart**

**Suppl. Fig. 7. Ex vivo gamma counting of [^64^Cu]CD19-mAb biodistribution showing no significant differences between EAE and naive heart, liver, and muscle 24 h after tracer injection.** *n*=5 naïve, *n*=6 EAE,***p*=.0021, *****p*<.0001, unpaired t-test.

## B220 Cell counting

**Cerebellum/meninges**

**Brainstem/meninges**

**Suppl. Fig. 8. Quantitative immunohistochemical quantitation of B220^+^ cell load in cerebellum and brainstem** *n*=5 naïve, *n*=10 EAE,**p*=.0195 for cerebellum, **p*=.0417 for brainstem, unpaired t-test. Brainstem was missing in one EAE mouse following tissue preparation and sectioning (*n*=9).

## Whole-body PET/CT images of tracer uptake in diseased and naïve animals

**Suppl. Fig. 9.** PET/CT images acquired 20 h post-injection, showing uptake of tracer in a) cervical lymph node, b) spleen, c) mesenteric lymph node and d) inguinal lymph node. Percentage of injected dose per gram (%ID/g) scaled to allow comparison between different mice.


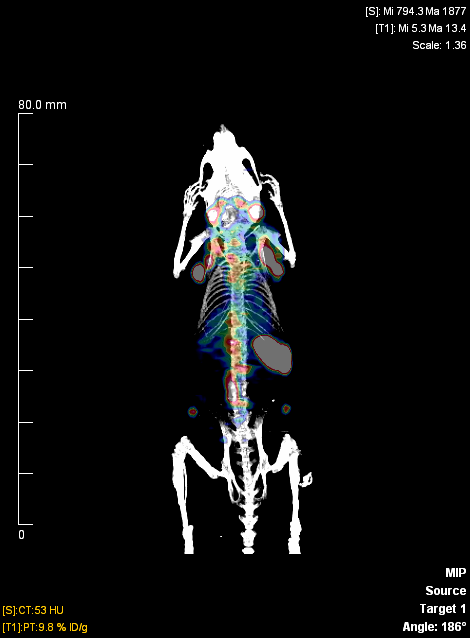

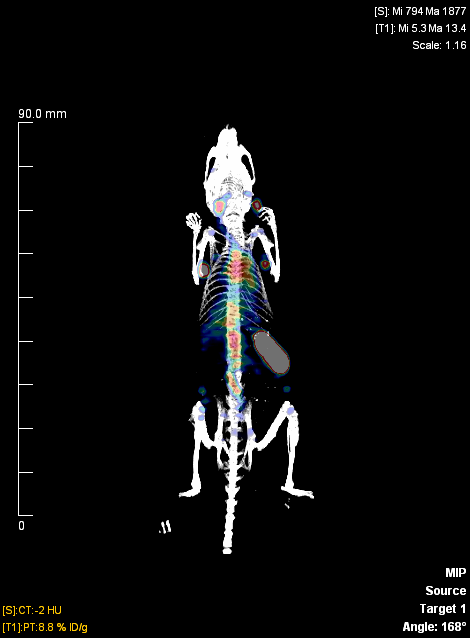

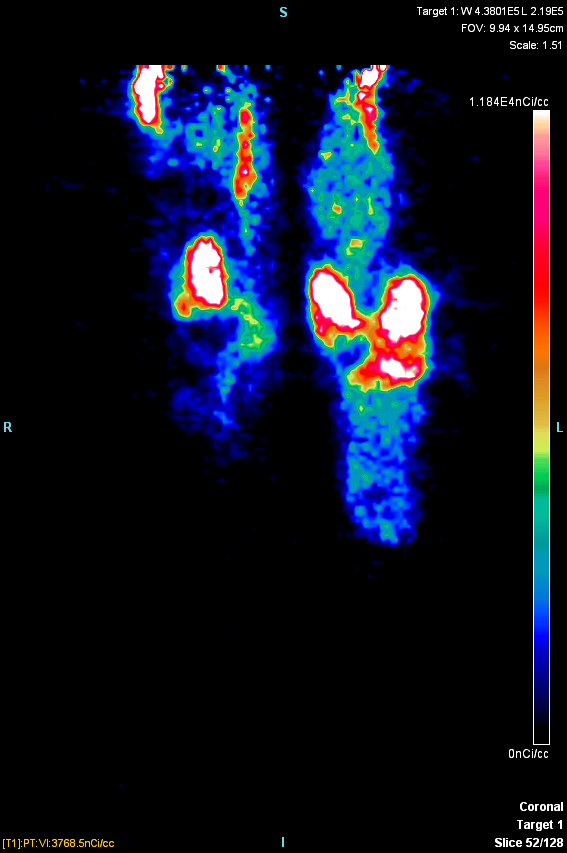


5

%ID/g

13

**EAE**

**Naive**

a)

b)

c)

d)

d)

c)

b)

a)
